# Characteristics of Beef Patties Substituted by Different Levels of Textured Vegetable Protein and Taste Traits Assessed by Electronic Tongue System

**DOI:** 10.3390/foods10112811

**Published:** 2021-11-15

**Authors:** Allah Bakhsh, Se-Jin Lee, Eun-Yeong Lee, Young-Hwa Hwang, Seon-Tea Joo

**Affiliations:** 1 Division of Applied Life Science (BK21 Four), Gyeongsang National University, Jinju 52852, Korea; drbakhsh01@gmail.com (A.B.); sejinlee1994@gmail.com (S.-J.L.); ley9604@gmail.com (E.-Y.L.); 2 Institute of Agriculture & Life Science, Gyeongsang National University, Jinju 52852, Korea; philoria@hanmail.net

**Keywords:** textured vegetable protein, plant-based ingredients, beef, taste traits, fatty acid

## Abstract

The main objective of this study was to incorporate soy-based textured vegetable protein (TVP) into beef patties in different quantities (10–40%) and compare various characteristics of these innovative formulations with a regular beef patty as a control. Incorporation of 10–40% TVP resulted in significantly lower (*p* < 0.05) moisture and fat contents, while higher crude fiber contents were detected compared to beef as the control. In addition, cooked patties showed higher pH levels (*p* < 0.05), with color coordinates expressing lighter, yellowish, and slightly redder indices than raw patties. Similarly, a plant protein that includes TVP minimizes (*p* < 0.05) WHC (water holding capacity), both RW% (release water) and CL% (cooking loss). Furthermore, hardness, cohesiveness, and thickness were reduced significantly (*p* < 0.05), while gumminess and chewiness increased (*p* < 0.05) considerably with the substitution of TVP (10–40%) compared to the control. Patties made without TVP received higher scores for sourness, bitterness, umami, and richness than the rest of the formulations. However, a higher tendency was detected for sourness, astringency, umami, and saltiness values with increasing additions of TVP. Nevertheless, hierarchical clustering revealed that the largest group of fatty acid profiles, including palmitoleic acid (C16:1), stearic acid (C18:0), and palmitic acid (C16:0), was slightly reduced with the addition of TVP, while arachidic acid (C20:0), lauric acid (C12:0), and oleic acid (C18:1) increased moderately with increasing levels of TVP. Meanwhile, the second-largest cluster that included linoleic acid (C18:2), arachidonic acid (C20:4), and linolenic acid (C18:3) increased enormously with higher levels of TVP incorporation. Taken together, it is suggested that incorporation of TVP up to 10–40% in beef patties shows promising results.

## 1. Introduction

Meat is an important dietary source of protein and several essential nutrients [1]. However, overconsumption and consumption of processed meat are risk factors for some forms of cancer, cardiovascular disease, and type 2 diabetes [2,3]. The potential reasons for these health concerns are the presence of an excessive volume of saturated fatty acids, specifically palmitic and myristic acids, their deleterious effects, and the oxidation of polyunsaturated fatty acids and synthetic stabilizers, which are known as potential health hazards [4]. Consequently, a trend is developing worldwide to reduce the consumption of red meat in the daily diet [5]. As part of this trend, flexitarian consumers expect that consuming plant-based products will help them reduce the intake of saturated fats and cholesterol associated with meat consumption [6].

Non-meat proteins are frequently used in the production of meat products to improve the texture and stability of the emulsion, reduce cooking losses, increase yield, and reduce costs [7]. During 1960s, TVP was invented and was subsequently used in meat analogs as a prime ingredient. TVP was allowed for use as the main ingredient for vegan versions of meat-based dishes, such as burgers and patties, with as much as 30% of the meat replaced with TVP [8]. The newest versions of meat analogs, e.g., Impossible Foods and Beyond Meat, have similar structures, comparable smells, and even a bloody appearance to help mimic animal meat, which has proven to be popular among consumers of meat substitutes with TVP added as the main ingredient. TVP is recognized as an important ingredient for its ability to contribute to two top food trends, including the continued quest for high-quality, low-fat foods and the thriving field of functional and nutraceutical foods [9]. TVP-based foods are often regarded as a healthy choice because they are cholesterol-free, low in fat, and low in calories [3].

The structure and texture of TVP can be modified by varying extrusion methods. TVP absorbs water and some fat and therefore has a physical function in addition to providing meat-like textural properties. It is widely used as an ingredient in ground meat for patties, sausages, and vegetarian foods and stews [10]. TVP is typically described as defatted soy flour or concentrates, mechanically treated by extruders to obtain chewy and meaty textures when rehydrated and cooked [11]. Previously, it has been stated that the integration of TVP in beef patties can reduce hardness, cohesiveness, and toughness [12]. However, the only incorporation of TVP cannot maintain the quality of the patty. Therefore, additional ingredients have been added to reduce complications associated with patty development. The flexible temperature of 25 °C for coconut oil supplies saturated fats that help make a typical patty [13].

Furthermore, methylcellulose is a cross-linker and binding agent that can be added to meat products, such as patties and burgers, due to its gelation capacity for solubility and its emulsifying characteristics [14]. Additionally, salt is used in the patty formulation to reduce cooking loss, intensify solubility, and help the patty retain moisture. Black pepper is also added to improve the breakdown and digestion of fats and proteins [15]. To date, limited research has been carried out to develop TVP-based products. Nevertheless, previous studies in the literature have looked at the incorporation of textured soy protein, corn starch, vegetable oil, salt, and soy protein isolate in typical meat-free products [16].

TVP and plant-based ingredients can therefore be used as substitutive ingredients in beef patties. However, the resultant products should retain the maximum similarity to the original products in terms of sensory qualities, textural acceptability, and satisfactory nutritional value [4]. Therefore, the main objective of the current study was to produce beef patties incorporating different levels of TVP and to measure the proximate, physicochemical, mechanical, and sensory characteristics of these patties relative to a control.

## 2. Materials and Methods

### 2.1. Materials

The formulas for the preparation of beef patties with different levels of TVP substituted are shown in Table 1. TVP (Anthony’s Goods, Glendale, CA, USA) was selected as the main substitute in beef patties (at different levels), and methylcellulose (high viscosity, Modernist Pantry, Eliot, ME, USA) was incorporated as the binder. Other ingredients, including molasses, yeast seasoning, umami seasoning, coconut oil, canola oil, beet juice, garlic powder, black pepper, and STPP, were used as described in Table 1. Round beef steaks were obtained from regional supermarkets in Jinju, Korea.

### 2.2. Sample Preparation and Processing

**Figure 1:**
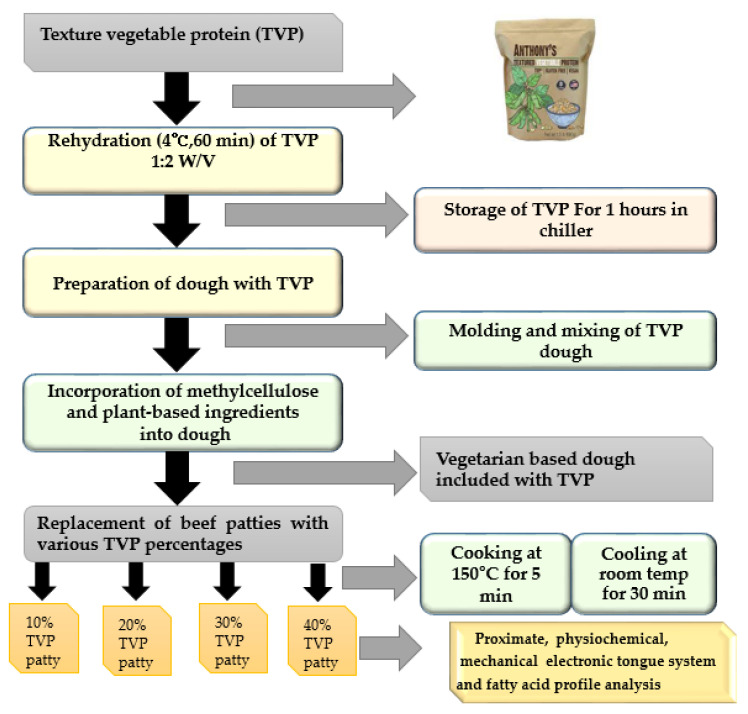
Flow diagram for the manufacture of beef patties with different levels of TVP substituted.

The flow diagram of the beef patties with different levels of TVP substituted is shown in Figure 1. TVP was incorporated as an essential ingredient in beef patties. The hydration procedure of TVP was adopted from Bakhsh et al. [14]. Consequently, according to each formulation, the specific amount of TVP was hydrated by soaking in cold water (at a ratio of 1:2) and stored in a chilling room for 1 h at 4 °C. The mixing and grinding process were adopted from Samard et al. [15] with slight modifications. Beef samples were minced by passing them through a meat grinder (Magimix Compact 3100, Montceau-Les-Mines, France) equipped with a 3 mm plate grinder. TVP was substituted in beef patties according to the individual formulation at various levels (10, 20, 30, and 40%) and mixed for 10 min with the rest of the ingredients listed in Table 1 using a Kitchen Aid mixer (Classic Plus Stand Mixer, St Joseph, MI, USA) until a homogenous distribution was observed.

**Figure 2:**
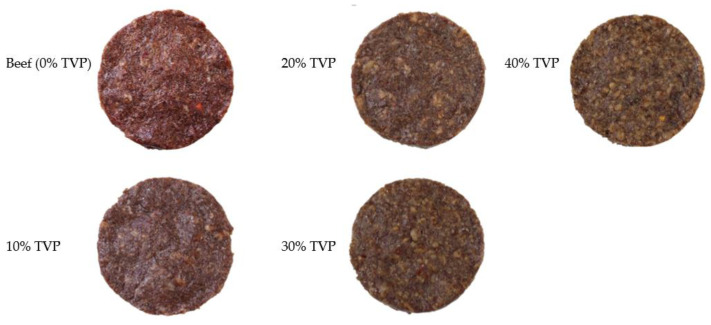
Typical beef patties with different levels of TVP substituted.

For each specification, 90 g of the mixture was then shaped into patties using a patty press maker (Figure 2). Finally, a beef patty including plant-based ingredients and without TVP was used for the control, as described in Table 1. Five formulations of beef patties varying in their TVP levels, with two batches of each (raw and cooked, *n* = 2), were used in the current experiment. A total of thirty patties were prepared and fifteen patties were allocated to each raw and cooked batch separately.

Through dry heat, the cooking procedure of patties was carried out on a non-stick pan (DWR-SH2000, Daewoo Maixingman Home appliances Co., Ltd., Gwangju, Korea) at 150 °C for 5 min on each side. Subsequently, the patties were flipped on a nonstick pan until the internal temperature touched 75 °C, as measured by a probe thermometer. The patties were allowed to cool at room temperature for 30 min before measuring the quality characteristics of the beef patties. Subsequently, physicochemical properties were assessed for both raw and cooked patties. Similarly, proximate composition, taste attributes, and fatty acid profile analysis were analyzed for the raw patties. However, mechanical property analyses were performed with the cooked patties.

### 2.3. Proximate Analysis

Based on the standard established procedure of AOAC [17], moisture, protein, and ash content were determined. Moisture content was measured in the oven (BioFree, BF-150C, Buchen, Korea) by dehydrating 5 g samples at 105 °C for 16 h. Protein determination was carried out using the established procedure of the Kjeldahl assay (B-324, 412, 435, and 719 S Titrino, BUCHI, Flawil, Switzerland) (N × 6.25).

$$\%N=\frac{\left[V\;\left(1\right)-V\;\left(B1\right)\right].F.c.F.M\;\left(N\right)\times 100}{M.1000}$$

% P = % N × PF

where V(1) represents the consumption of titrant, sample (ml); V(BI), the average consumption of titrant, blank (ml); F, the molar reaction factor (1 = HCl, 2 = H_2_SO_4_); c, the concentration of titrant [mol/L]; M(N), the molecular weight of N (14.007 (g/mol); M, the sample weight (g); 1000, the conversion factor (ml in L); and PF, the protein factor.

The crude fat content was determined using the procedure established by Folch et al. [18], with slight modifications. Briefly, 3 g of the sample was homogenized with a homogenizer (IKA T25 ULTRA-TURRAX, Staufen, Germany), and 30 mL of already prepared Folch solution I (chloroform: methanol = 2:1, *v/v*) was added to the subsequent sample to extract the lipids. Using Whatman No. 1 filter paper, the solution, including the sample, was filtered into a graduated cylinder; afterward, the solution was stirred with 0.88% NaCl to separate it into two distinct layers in the cylinder. Using 10 mL of Folch solution II (chloroform:methanol:H_2_O = 3:47:50), the graduated cylinder was washed thoroughly, and the maximum volume of the lower meniscus was observed in the graduated cylinder. Then, using an aspirator, the upper layer (methanol and water layer) was sucked and discarded. The lower layer (chloroform containing lipid extracts) was poured into the AL plate and dried at 50 °C in a dry oven. Through electrical balance, the weight of the dish was measured before and after drying, and, subsequently, the crude fat content was determined through the weight difference of the dish.

The determination of crude fiber was estimated using an Ankom 200 fiber analyzer (Ankom Technology, Macedon, NY, USA) by digesting 0.5 g with H_2_SO_4_ and NaOH. Weight loss resulting from ashing (2 h at 600 ± 15 °C) was determined to calculate the crude fiber content [19]. The crude fiber content was calculated using the following formula:

$$\mathrm{Crude}\;\mathrm{fiber}\;\%\;=\frac{\left[W3-\left(W1\times C1\right)\right]}{W2}\times 100$$

where W1 = bag tare weight, W2 = sample weight, W3 = weight of organic matter (loss of weight on ignition of bag and fiber), and C1 = ash corrected blank bag factor, (loss of weight on ignition of blank bag/original blank bag).

### 2.4. Physicochemical Analysis

The pH of raw and cooked patties was measured using a portable pH meter (Mettler Toledo, MP 230 Schwerzenbach, Switzerland). With the homogenizer (IKA T25 ULTRA-TURRAX, Staufen, Germany), 3 g samples from each patty sample were homogenized at a speed ranging from 3000–25,000 rpm, with 27 mL of deionized water for 30 s. The calibrated pH meter (7.00, 4.01, and 9.21) was used for the evaluation of pH.

The color coordinates of raw and cooked patties were measured using a Konica Minolta Colorimeter (Chroma meter, CR-300, Tokyo, Japan) using an established method described by Bakhsh et al. [14]. The instrument was equipped with a standard D65 illuminant using a 2° position of the standard observer with a pulse xenon lamp and an 8 mm reading surface area. To determine color coordinates, the Minolta Chromameter calorimetric instrument (CR -300, Minolta, Tokyo, Japan) was calibrated using a white ceramic plate (Y = 93.5, X = 0.3132, y = 0.3198) and from various locations of sample lightness values (L*), redness (a*), and yellowness (b*).

Released water (RW%) was measured based on a method described by Joo [20]. Briefly, each patty sample (3.0 ± 0.05 g) was placed on a previously dried and weighed filter paper (Whatman No. 1, 11 cm diameter) with two thin plastic films. After weighing them, the filter paper and plastic film with the meat samples were positioned between Plexiglas plates. A load of 2.5 kg and free mechanical force were applied for 5 min. Then, the wet filter papers and plastic films were quickly weighed after carefully removing the compressed meat. Using the following formula, RW% values were calculated:

$$\mathrm{RW}\;\%\;=\frac{\left[\mathrm{Damp}\;\mathrm{filter}\;\mathrm{paper}\;\mathrm{and}\;\mathrm{plastic}\;\mathrm{weight}\;-\mathrm{Filte}\;\mathrm{paper}\;\mathrm{and}\;\mathrm{plastic}\;\mathrm{films}\;\mathrm{weight}\right]}{\mathrm{raw}\;\mathrm{patty}\;\mathrm{weight}\;\left(g\right)}\times 100$$

The cooking loss (CL%) was determined as a percentage using a method described by Bakhsh et al. [14]. Briefly, for CL%, a cut was made in the middle of each patty with dimensions of 4 × 3 × 3 cm (L × H × W) and the samples were packed in a low-density polyethylene bag. Samples were cooked at 75 °C for 30 min and chilled immediately in ice flakes for 10 min. CL% was determined by weight difference before and after cooking using the following formula:

$$\mathrm{CL}\;\%\;=\frac{\left[\mathrm{raw}\;\mathrm{patty}\;\mathrm{weight}\;\left(g\right)-\;\mathrm{cooked}\;\mathrm{patty}\;\mathrm{weight}\;\left(g\right)\right]}{\mathrm{raw}\;\mathrm{patty}\;\mathrm{weight}\;\left(g\right)}\times 100$$

### 2.5. Mechanical Properties

The texture profile analysis (TPA) of the cooked patties was performed using a Sun Rheometer (Compact-100 II, Sun Scientific Co., LTD., Tokyo, Japan). The patties were evenly designed (20 mm in height and 26 mm in diameter), and compressed in axial orientation through a rheometer with a flat pressure adapter (No. 1). The samples were compressed at a crosshead speed of 60 mm/min at a final strain of 60% through a two-cycle sequence with a load cell of 10 kg [21]. The following parameters were estimated: hardness, cohesiveness, springiness, gumminess, and chewiness.

The shrinkage percentage was determined with the following formula:

$$\mathrm{Shrinkage}\;\left(\%\right)=\frac{\left[\mathrm{raw}\;\mathrm{patty}\;\mathrm{diameter}\;\left(\mathrm{mm}\right)-\;\mathrm{cooked}\;\mathrm{patty}\;\mathrm{diameter}\;\left(\mathrm{mm}\right)\right]}{\mathrm{raw}\;\mathrm{patty}\;\mathrm{diameter}\;\left(\mathrm{mm}\right)}\times 100$$

The percentage of thickness change of the patties was measured at four different locations of the raw and cooked patties. The percentage thickness change of the raw and cooked patties was determined by the following formula:

$$\mathrm{Thickness}\;\mathrm{change}\;\left(\%\right)=\frac{\left[\mathrm{raw}\;\mathrm{patty}\;\mathrm{thickness}\;\left(\mathrm{mm}\right)-\;\mathrm{cooked}\;\mathrm{patty}\;\mathrm{thickness}\;\left(\mathrm{mm}\right)\right]}{\mathrm{raw}\;\mathrm{patty}\;\mathrm{thickness}\;\left(\mathrm{mm}\right)}\times 100$$

### 2.6. Taste Attributes Determination by the Electronic Tongue System

For the taste attributes of the beef patty with TVP substituted, the electronic tongue system (ETS) (Intelligent Sensor Technology, SA402B, Kanagawa, Japan) was adopted using a procedure established by Ismail et al. [22], with minor modifications. The ETS system comprises various sensor arrays, including reference and lipid/membrane electrodes and a data analysis software package, and is equipped with unique artificial lipid membranes to quantify possible deviations due to the adsorption of ingredients linked to dissimilar taste attributes.

The ETS system is made up of five different taste sensors. Based on taste determination traits, the sensor was named CA0 (to detect sour substances), AAE (to detect umami substances), C00 (to detect bitter substances), AE1 (to detect astringent substances), and CT0 (to detect salty substances), respectively. Before analysis, the sensors were equipped with a solution (reference solution, 30 mm KCl solution comprising 0.3 mm tartaric acid). Subsequently, the minced patty (90 g) was allowed to be mixed with deionized H_2_0 (400 mL). The extraction process was carried out for 10 min at 85 °C, followed by centrifugation at 3000× *g* for 10 min. The formation of supernatant due to centrifugation was filtered with Whatman No. 1 filter papers, and, subsequently, the resultant supernatant was shifted for further investigation. 

Control for the beef patties with TVP substituted was considered standard (0) in the current analysis. Before examining the taste traits of patties, the membrane potential (Vr) was first verified for the sensors concerning the reference solution, tracked by a measurement (Vs) in the sample solution. Furthermore, the sensor was cleaned with caution using the reference solution, and the potential (Vr0) was documented.

### 2.7. Fatty Acid Profile

As previously described in the fat determination method, the extraction of lipids from patties replaced with TVP was adopted from Folch et al. [18]. Subsequently, through the saponification process, lipid methyl esters were determined by adding 1.0 N methanolic NaOH, and immediately afterward the samples were methylated with boron trifluoride in methanol.

A permanently stationed gas chromatograph HP6890N (Hewlett–Packard, Santa Clara, CA, USA) attached to an automatic sampler HP7683 (Hewlett–Packard) was used to analyze fatty acid methyl esters (FAME). FAME separations were carried out using a 100 m SP2560 (Supelco, Santa Clara, CA, USA) equipped with a capillary column (0.25 mm id and 0.20 μm film thickness).

A temperature database with nitrogen as the carrier gas at a flow rate of 1 mL/min was applied to purify FAME from samples. The chromatograph holding column oven temperature increased by reducing time, i.e., 50–180 °C at 10 °C per min, 180–220 °C at 5 °C per min, 220–240 °C at 2 °C per min, and then held at 240 °C for 20 min, respectively. The evaluation of the samples was carried out by two replications with 1 L of sample injection of 1 μL. Identification of individual fatty acids was made by the association of retention times with standards (Supelco 37 components FAME Mix, Santa Clara, CA, USA). The results were stated as the percentage of total fatty acids observed in the total peak area of the respective fatty acids.

### 2.8. Data and Statistical Analysis 

Before analysis, Shapiro Wilk and Levene’s tests were used to assess the distribution’s normality and the equality of variances. All percentage records below 20% and above 80% were transformed by the square root of the arcsine. Data were statistically analyzed using one-way analysis of variance (Factorial ANOVA) using SPSS version 23 (IBM Corp., Armonk, NY, USA). Tukey’s post hoc test was performed following a significant *p*-value to assess differences between means. Data in the text are given as the mean ± standard error of the mean (SEM). A *p*-value ≤ of 5% was considered significant. Results are expressed as least-squares mean values of three independent replications. A hierarchical cluster tree was constructed based on the relative composition of fatty acids using the package ’ComplexHeatmap’ with the R software version 4.0.3 (R Core Team, 2020). Finally, Pearson’s correlations between the fatty acid composition and the ETS were applied to develop a correlation matrix using the ‘CORR procedure’ of the SAS software.

## 3. Results and Discussion

### 3.1. Proximate Analysis

The addition of TVP as a substitute significantly influenced the chemical composition of the beef patties (Table 2). The partial substitution of beef patties with TVP (10, 20, 30, and 40%) resulted in substantially lower moisture, fat (*p* < 0.05), and higher crude fiber contents among all formulations. The moisture content of the patties with TVP substituted ranged from 65.18 to 51.32% and was considerably (*p* < 0.05) affected by TVP incorporation. The higher the volume of TVP substituted in the beef patties, the less moisture was detected. In contrast, a reverse trend was observed for the crude fiber content; it improved significantly (*p* < 0.05), with higher volumes of TVP assimilated. Among the formulations, the level of fat and crude fiber content ranged from 4.39 to 3.46% and 1.0 to 1.97% with the incorporation of TVP (0–40%), respectively. Furthermore, increased incorporation of TVP resulted in a linear and quadratic increase in ash and crude fiber, while reducing moisture.

The low values in fat content with the addition of TVP are due to a high-pressure extrusion process during the formation of TVP. The extrusion process eliminates fat with nonpolar solvents, such as hexane, and requires low/intermediate moisture to form TVP from soy protein [3]. The higher fiber content and lower moisture were reported in meat analog patties produced with commercial TVP and textured-isolate soy protein, and in light pork burgers made with soy protein isolate, with beef and pork as control, respectively [14,23]. As TVP is a fibrous soy-based protein, a higher amount of TVP incorporation ultimately leads to more fibrous patties, from 10 to 40% substitutions. Furthermore, variations in chemical composition, including moisture, fat, and fiber content in different formulations, could be the basis for the vast differences in physicochemical characteristics that might occur due to the substitution of beef with TVP [11]. Inconsistent with the current study, it has been confirmed that lower moisture content in soy-based patties caused an increase in protein and lipid indices [24]. Moreover, Lin and Mei [25] and Danowska [26] reported similar findings in low-fat meat batters and pork patties with low-fat soy protein added, respectively. On the other hand, goat meat patties with 15% incorporated soy paste did not affect moisture, fat, or protein content [27].

### 3.2. Physiochemical Properties 

The physicochemical properties of the beef patties with TVP substituted at different levels are shown in Table 3. In raw patties, beef patties with TVP do not show any variation in pH values (*p* > 0.05) among various formulations. However, cooked patties substituted with (0 to 40%) TVP confirmed significantly higher (*p* < 0.05) pH attributes (*p* < 0.05) (6.11 to 6.46) among all formulations, respectively. The higher trend in pH among treatments with the incorporation of a higher level of TVP could be due to the slight alkalinity of TVP (pH 7.42–7.43) [28]. Previously, Bakhsh et al. [14] reported higher pH levels in cooked plant-based meat patties with the addition of two different TVPs at different levels of methylcellulose. In cooked patties, the higher pH might be due to heated imidazolium, the basic R group of the amino acid histidine, which could be exposed during heating [29].

Substituting beef with TVP slightly reduces the L* values in raw patties, although the L* values increase significantly in all formulations in cooked patties. The lower tendency in L* values with the incorporation of raw TVP was previously observed by Samard and Ryu [30], and they also confirmed that the decline in L* values might be due to the extrusion process, caramelization, and hydrolysis, as well as the degradation of pigments. In a similar pattern, the b* values in the raw patties did not show any difference, although b* values in the cooked patties increased significantly in all formulations. The possible variations in color coordinates in cooked patties might be due to the heating process, which influences the color of meat products due to the denaturation and oxidization of three forms of myoglobin (oxyMb, deoxyMb, and metMb) [30]. In addition, a linear and quadratic increase was detected in pH, L*, and b* with higher incorporations of TVP. The increment in the L* value in cooked patties with an increased level of substitution might be associated with the natural pigmentation of meat and legumes, such as soy, which contains certain amounts of symbiotic hemoglobin known as leghemoglobin [31], which can cause substantial variations in color coordinates according to various levels of TVP substitution in beef patties. Several lipid oxidation products, e.g., malonaldehyde, will enhance meat discoloration and cause an increase in L* values and a decrease in b* values. Similarly, the yellowish coloration of beef patties incorporating TVP could be due to the yellowish color of TVP-based soy protein [3]. The color coordinates (L* and b*) were positively affected after cooking (CL%) in the case of the patties with various levels of TVP incorporated. However, RW% showed no relation to color coordinates in raw patties as compared with those with different levels of TVP incorporated.

The capability of retaining water and other juices in the patty before and after cooking is an essential product characteristic. A trend of decrement (*p* < 0.05) was observed in WHC, including RW% and CL%, with the substitution of beef with TVP from 10 to 40%. In addition, higher TVP incorporation resulted in a linear and quadratic decrease in RW% and CL%. The current results were in line with Hale et al. [32], who stated that patties made with textured soy protein showed a lower WHC than a beef patty as a control. Similarly, the lower cooking loss was reported in goat meat patties incorporated with textured soy protein [33]. Similarly, a negative relationship was detected between textured soy protein and cooking loss in cooked hamburger patties in both linear and quadratic patterns [34]. Moreover, WHC depends on protein composition, protein denaturation, as well as the extent of interaction with water and oil [35]. The significant differences in CL% among various beef patties made with TVP could be attributed to a rehydration effect of the soy protein during patty formulation.

### 3.3. Mechanical Properties 

A decrement trend was recorded for the cohesiveness of the hardness and thickness of the beef patties with TVP substituted from 10 to 40%. Contrary to this, gumminess and chewiness increased significantly (*p* < 0.05) among all TVP replacement formulations for beef patties (Table 4). In addition, a linear and quadratic decrease in hardness and cohesiveness was detected, while springiness and chewiness increased with higher incorporations of TVP. Previously, Kamani et al. [4] reported that sausage samples containing more meat require a greater grinding force than meatless samples containing plant-based ingredients. Therefore, the decline in hardness and cohesiveness of the beef patties substituted with TVP (10–40%) could be due to a weaker myofibril protein network, which reduced the resistance of the product to compression. Likewise, based on interactions between myofibrillar and soy protein, there is strong evidence for less stable conformations, which eventually compromise the texture of the final product [36]. In line with the current study, Samard et al. [15] reported similar findings relative to hardness and cohesiveness, as well the cutting strength of the fully incorporated TVP burger patties, which was impressively reduced compared to that of the control patty. Furthermore, our previous study also confirms our current findings, with the hardness, chewiness, and gumminess of the control being significantly higher compared to the C-TVP and T-ISP of meat analog patties, respectively [14]. The beef patty control exhibited greater hardness and a firmer texture due to muscle protein denaturation in the meat system [37].

The shrinkage and percentage of the thickness of the beef patties made with TVP were significantly reduced (*p* < 0.05) as the level of TVP increased from 10 to 40%. Previously, incorporating a higher percentage of textured soy protein was reported to lead to shrinkage in goat and beef patties after cooking [33,38]. Furthermore, a negative relationship was found between increased TVP incorporation and shrinkage in meat analog patties with different levels of methylcellulose concentrations [14]. Therefore, the higher percentage shrinkage and reduction in thickness are probably caused by the denaturation of proteins through the cooking procedure, which ultimately enhances the release of extra fluid in meat compared to beef patties made with TVP.

### 3.4. Sensory Evaluation by Electronic Tongue System 

The ETS has been an established procedure in our research facility. We have previously successfully determined the taste characteristics of low-temperature and long-time single and two-stage sous-vide cooking methods [22] and the flavor characteristics of chicken nuggets with milk fat added [39]. Furthermore, we investigate for the first time the evaluation of taste traits in terms of sourness, bitterness, astringency, umami, richness, and saltiness for beef patties made with TVP (Table 5). In this part, the control was represented as standard, and its taste characteristics were calculated as 0 by the sensor response output. Among the five sensors used, significant differences were found in the response to all tastes as the various proportions of beef were replaced with TVP; a tendency to increase was observed for the sourness, astringency, umami, and saltiness measures as greater quantities of TVP replaced beef in the patties. However, the bitterness attribute showed a negative trend for all levels (10–40%) of beef patties made with TVP except for the 0% patty. Nevertheless, based on TVP replacement in beef patties, richness values were significantly reduced as the added TVP increased from 10 to 40%. Additionally, the bitterness decreased and the saltiness increased quadratically, while a linear and quadratic decrease was detected in the bitterness, umami, and richness with higher TVP incorporations. 

In line with current results, it has been reported that sourness, saltiness, and bitterness increased in fermented sausages as the storage time increased [40]. The possible reason for the increase in sourness might be the generation of acid during the metabolism of carbohydrates in meat and TVP. Similarly, astringency and bitterness are associated with tannins derived primarily from plant ingredients [41]. Consequently, higher levels of TVP, such as soy protein (10–40%) and some shared elements, particularly spices, garlic powder, and black pepper used in the preparation of patties, could be responsible for higher astringency and bitterness. However, the main source that causes differences in astringency between patties with different levels of TVP concentration is not entirely clear. As expected, the umami taste increased with a higher TVP level, with the maximum umami score found at 0% (1.74) and the second-highest score at 40% (0.80). Our study shows objectively decent similarity to the findings with umami taste scores (0.63–2.50) of stewed chicken evaluated by an electronic nose system [42]. Although taste is a key issue for beef patties made with TVP, only limited studies have highlighted umami, for example, in pork [43] and beef [44]. Regarding the richness and saltiness previously reported by Sabuikun et al. [39], similar results were reported for chicken nuggets with increasing saltiness values as the level of milk fat and mashed potato increased. Therefore, further sensory evaluation with ETS of beef patties made with different quantities of TVP becomes difficult due to limited or no related information.

### 3.5. Fatty Acid Profile

**Figure 3:**
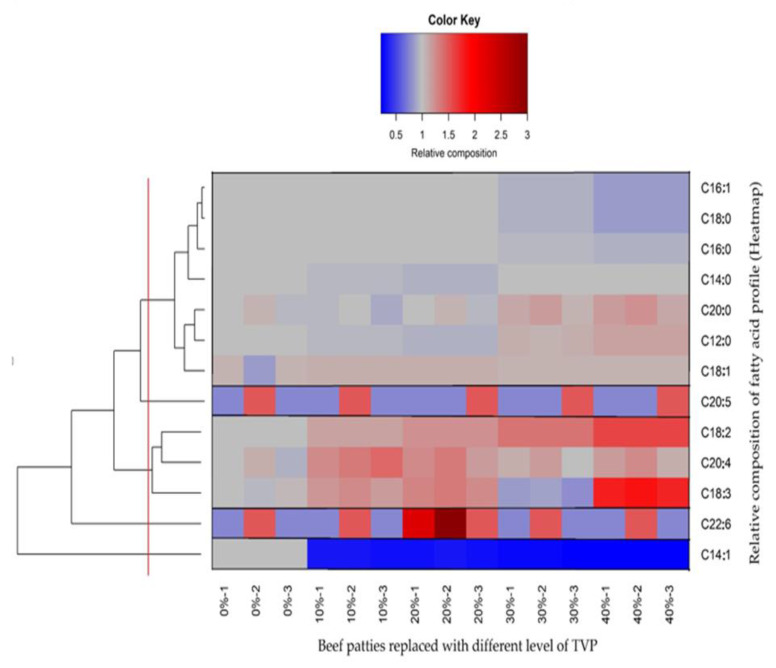
Relative composition of the fatty acid profile with the incorporation of different levels of TVP (Heatmap).

The relative fatty acid composition data were clustered into five different groups according to Euclidian distance (Figure 3). As is evident from the clustering, the percentage of most fatty acids was significantly affected by the addition of TVP. Two main clusters could be revealed: the first and most significant was composed of fatty acids C16:1, C18:0, C16:0, myristic acid (C14:0), arachidic acid (C20:0), C12:0, and C18:1. In the mentioned group, the relative composition of fatty acids, such as C16:1, C18:0, C16:0, was slightly reduced (light blue color), while C20:0 C12:0 and C18:1 increased moderately (light red colors) with increasing TVP levels (30–40%) respectively. Previously He et al. [1] reported that commercial plant-based burger 3 and plant-based burger 4 had similar configurations of fatty acids C18:0 and C16:0, which reduced significantly with the addition of textured soy protein. In contrast, C14:0, C20:0, and C12:0 showed no difference compared to plant-based burger 1 and plant-based burger 2, respectively. Furthermore, in accordance with current findings, fatty acids C16:1, C18:0, and C16:0 were significantly reduced in low-fat burgers containing Inulin and β-glucan as compared to beef burgers [45]. They further explained that the main significant difference in fatty acid concentrations in different formulations was due to the substitution of animal fat for pre-emulsified canola oil, which ultimately supports our findings, as we added canola and coconut oil in the present study (Table 1). The second part of the first group contains medium-chain fatty acids (C20:0, C12:0, and C18:1), which expressed slightly higher activity with a higher level of TVP incorporation; this could be due to the presence of an oil ingredient in the patties samples—coconut oil naturally contains a high concentration of medium-chain fatty acids [46]. Inconsistent with the present study, it was stated that plant-based burgers had higher medium-chain fatty acids than meat-based burgers [5].

The second larger group included C18:2, C20:4, and C18:3, which increased moderately to a strong level with a higher level of TVP incorporation of 10 to 40%. Why incorporating a higher level of TVP causes higher values of C18:2, C20:4, and C18:3 is still unclear. However, previous studies were conducted to support the fatty acid profile of the current study with a long-chain fatty acid, C16:0, C18:0, oleic acid (C18:1), C18:2, and C18:3 were the main fatty acids found in plant-based meat analogs with the addition of canola oil [47]. Furthermore, the fatty acid composition of the beef patties made with TVP was previously described in sausages prepared with pork back fat. The main saturated fatty acids were C16:0 and C18:0, the main monounsaturated fatty acid was C18:1, and the main polyunsaturated fatty acids were C18:2 and C18:3. On the other hand, eicosapentaenoic acid (C20:5), docosahexaenoic acid (C22:6), and myristoleic acid (C14:1) were the only fatty acids in their groups [48]. A group included with a single fatty acid C14:1 expressed a strong tendency toward decrement (dark blue color), starting from the minimum level of TVP incorporation to the highest level. The previous literature on C14:1 contradicts our results, with no differences between commercial plant-based burgers and a similar trend reported in pre-cooked legume-based burgers [1,49].

### 3.6. Correlation Coefficient (r) between ETS and Fatty Acid Profile 

**Figure 4:**
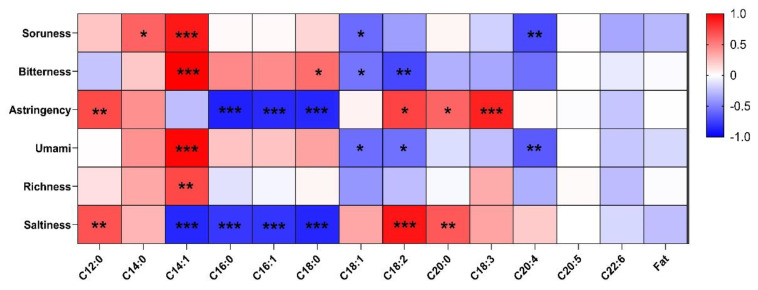
The correlation coefficient between the fatty acid profile and the electronic tongue system. * p < 0.05, ** *p* < 0.01, *** *p* < 0.001.

Figure 4 shows the correlation between the fatty acid profile and the taste characteristics of the beef patties made with TVP. The bitterness of taste attribute expressed a strong positive correlation with C14:1 (r = 0.90 and 0.99, respectively) and negatively correlated with acids C20:4 and C18:2 (r = −072 and −0.72, respectively). Similarly, astringency was positively correlated with C12:0 and C18:3 (r = 0.71 and 0.87), while strongly negative correlations were found with C16:0, C16:1, and C18:0 (r = −0.88, −084 and −0.84), respectively. Furthermore, umami and richness attributes were positively correlated with C14:1 (r = 0.98 and 0.72, respectively). Additionally, saltiness showed a strong positive correlation with C18:2 and a positive correlation with C12:0 and C20:0 (r = 0.67 and 0.65); however, a strong negative correlation was recorded with C14:1, C16:0, C16:1, and C18:0 (r = −0.85, −0.79, −0.80, and −0.86) respectively. Few studies are available on the taste properties of beef patties made with TVP with respect to fatty acids. However, the taste quality and threshold fatty acid concentration, such as C12:0, C14:0, and C16:0, were described as irritants, metallic, and bitter [50], which corresponds closely to our results shown in Figure 4. The fatty acid C18:0 was previously designated as sour, bitter, and astringent, while C18:1 was metallic, astringent, and bitter [50,51]. Similarly, C18:2 retains the taste attributes of irritant, metallic, and bitter [50]. However, it was previously stated that the taste thresholds estimated for saturated fatty acids are directly proportional to the carbon chain length of individual fatty acids. A similar trend was observed for caproic (C6:0), (C12:0), and (C18:1) [52]. Regarding polyunsaturated fatty acids (PUFA), the average taste thresholds for lC18:2 and C18:3 were 5.6 and 2.5 times lower than C18:1 [51]. Regarding PUFAs of C20 and C22, such as eicosapentaenoic acid (EPA, C20:5, n-3) and docosahexaenoic acid (DHA, C22:6, n-3), only data on their effects on other perceptions of taste were available [53].

## 4. Conclusions

This study investigated the suitability of TVP as a partial substitute for beef in beef patties at various levels. The results demonstrated that incorporating a higher level of TVP improved the color coordinates, producing lighter and redder patties. In addition, the cooking had a positive effect on the physicochemical properties of the patties made with TVP compared to the control. The addition of TVP resulted in lower values for cohesiveness and hardness, with higher scores for gumminess and chewiness. Patties with the maximum level of TVP incorporation showed a detectable level of sourness, astringency, umami, and saltiness. Furthermore, the clustering indicated that most of the fatty acids were significantly affected by the addition of TVP. The general substitution of 10–20% TVP appeared closer to the control patty. However, these results suggest that TVP can be substituted up to 10–40% in beef patties without compromising patty quality characteristics compared to the control.

## Figures and Tables

**Table 1:** Treatment and formulation of beef patties replaced with different levels of TVP.

Ingredients %			Treatments		
			TVP %		
	0%	10%	20%	30%	40%
Ground beef	75.88	65.88	55.88	45.88	35.88
TVP	−	10.00	20.00	30.00	40.00
Methylcellulose	3.00	3.00	3.00	3.00	3.00
Garlic powder	2.25	2.25	2.25	2.25	2.25
Yeast extract	2.25	2.25	2.25	2.25	2.25
Black pepper	1.50	1.50	1.50	1.50	1.50
Mushroom	2.25	2.25	2.25	2.25	2.25
Salt	1.12	1.12	1.12	1.12	1.12
Coconut oil	3.75	3.75	3.75	3.75	3.75
Canola oil	3.75	3.75	3.75	3.75	3.75
Beet juice	1.50	1.50	1.50	1.50	1.50
Molasses	1.50	1.50	1.50	1.50	1.50
Smoked flavor	0.75	0.75	0.75	0.75	0.75
STPP	0.50	0.50	0.50	0.50	0.50

TVP: textured vegetable protein. STPP: Sodium tripolyphosphate.

**Table 2:** Proximate chemical composition of beef patties replaced with different levels of TVP.

Parameters	TVP (%)					*p*-Value		
	0	10	20	30	40	ANOVA	Linear	Quadratic
Moisture %	65.18 ± 0.45 ^ab^	59.00 ± 0.85 ^a^	53.10 ± 0.50 ^c^	52.73 ± 0.95 ^bc^	51.32 ± 0.80 ^bc^	<0.001	0.004	0.019
Ash %	3.16 ± 0.16 ^a^	3.98 ± 0.50 ^ab^	4.67 ± 0.43 ^b^	4.50 ± 0.28 ^b^	5.16 ± 0.35 ^b^	0.027	<0.001	0.005
Fat %	4.39 ± 0.15 ^c^	3.63 ± 0.99 ^b^	3.56 ± 0.25 ^a^	3.45 ± 0.99 ^ab^	3.46 ± 0.15 ^ab^	<0.001	0.952	0.614
Protein %	15.38 ± 0.45 ^ab^	16.42 ± 0.65 ^ab^	14.75 ± 0.25 ^a^	15.96 ± 0.66 ^ab^	17.32 ± 1.27 ^b^	0.212	0.195	0.224
Crude fiber %	1.00 ± 0.00 ^a^	0.99 ± 0.003 ^a^	0.99 ± 0.008 ^a^	1.66 ± 0.33 ^b^	1.97 ± 0.27 ^b^	0.002	<0.001	<0.001

^a–c^ Different superscript letters within the same row mean significantly different between treatments (*p* < 0.05). SEM: standard error of the mean; TVP: textured vegetable protein.

**Table 3:** Physiochemical characteristics of the beef patties replaced with different levels of TVP.

Parameters	TVP (%)					*p*-Value		
	0	10	20	30	40	ANOVA	Linear	Quadratic
**Raw patties**								
pH	6.05 ± 0.28	6.04 ± 0.23	6.02 ± 0.10	6.18 ± 0.14	6.13 ± 0.11	0.395	0.152	0.343
L*	48.35 ± 0.23 ^d^	47.24 ± 0.67 ^bc^	47.36 ± 0.66 ^bc^	45.63 ± 0.27 ^ab^	44.72 ± 1.17 ^a^	0.026	0.151	0.014
a*	5.58 ± 0.26	6.54 ± 0.39	6.56 ± 0.79	6.10 ± 0.26	6.26 ± 0.71	0.365	0.131	0.347
b*	10.27 ± 0.44 ^a^	10.30 ± 0.23 ^a^	10.35 ± 0.77 ^a^	8.92 ± 0.39 ^b^	10.40 ± 0.45 ^a^	0.006	0.082	0.019
RW %	8.16 ± 0.76 ^b^	5.17 ± 0.70 ^a^	4.01 ± 0.54 ^a^	3.68 ± 0.69 ^a^	3.86 ± 0.29 ^a^	0.002	<0.001	<0.001
**Cooked patties**								
pH	6.11 ± 0.15 ^a^	6.18 ± 0.01 ^b^	6.29 ± 0.21 ^c^	6.34 ± 0.02 ^d^	6.46 ± 0.006 ^e^	<0.001	<0.001	<0.001
L*	32.46 ± 0.69 ^a^	33.92 ± 0.73 ^ab^	35.80 ± 0.55 ^bc^	36.49 ± 0.81 ^d^	37.60 ± 0.321 ^d^	0.002	<0.001	<0.001
a*	6.11 ± 0.14	6.48 ± 0.11	6.5 ± 0.54	6.00 ± 0.37	6.54 ± 0.74	0.691	0.987	0.955
b*	12.79 ± 1.35 ^a^	14.00 ± 0.60 ^ab^	15.35 ± 0.81 ^ab^	16.56 ± 0.78 ^bc^	18.91 ± 0.12 ^c^	0.004	<0.001	<0.001
CL %	16.81 ± 0.93 ^c^	12.53 ± 1.02 ^bc^	6.86 ± 0.34 ^a^	10.61 ± 0.92 ^ab^	7.16 ± 2.93 ^a^	0.005	0.003	0.005

^a–e^ Different superscript letters within the same row mean significantly different between treatments (*p* < 0.05). SEM: standard error of the mean; TVP: Textured vegetable protein.

**Table 4:** Mechanical properties of beef patties replaced with different levels of TVP.

Parameters	TVP (%)					*p*-Value		
	0	10	20	30	40	ANOVA	Linear	Quadratic
Hardness (N)	32.95 ± 1.01 ^e^	26.01 ± 0.51 ^d^	20.26 ± 0.88 ^c^	15.84 ± 0.41 ^b^	12.51 ± 0.56 ^a^	<0.001	<0.001	<0.001
Cohesiveness	0.44 ± 0.12 ^c^	0.39 ± 0.18 ^ab^	0.41 ± 0.13 ^b^	0.37 ± 0.08 ^ab^	0.35 ± 0.18 ^a^	0.005	<0.001	0.004
Springiness (mm)	2.7 ± 0.22 ^a^	2.62 ± 0.30 ^a^	2.26 ± 0.60 ^ab^	3.15 ± 0.37 ^ab^	3.20 ± 0.24 ^ab^	0.731	0.002	0.008
Chewiness (mJ)	0.26 ± 0.15 ^a^	1.60 ± 0.26 ^b^	2.00 ± 0.50 ^ab^	2.52 ± 0.33 ^bc^	2.73 ± 0.80 ^c^	<0.001	<0.001	<0.001
Gumminess (N)	1.74 ± 0.19 ^ab^	1.83 ± 0.14 ^ab^	2.16 ± 0.37 ^b^	2.04 ± 0.19 ^b^	2.17 ± 0.26 ^b^	0.651	0.854	0.427
Shrinkage %	16.42 ± 0.35 ^a^	16.94 ± 1.87 ^a^	13.79 ± 1.08 ^a^	14.85 ± 0.90 ^a^	13.18 ± 0.71 ^a^	0.273	0.916	0.324
Thickness %	35.45 ± 2.92 ^bc^	24.39 ± 3.42 ^a^	25.59 ± 1.80 ^ab^	23.79 ± 0.87 ^c^	21.26 ± 1.15 ^ab^	<0.001	1.000	0.709

^a–e^ Different superscript letters within the same row mean significantly different between treatments (*p* < 0.05). SEM: standard error of the mean; TVP: Textured vegetable protein.

**Table 5:** Sensory evaluation of beef patties made with different levels of TVP by an electronic tongue system.

Parameters		TVP (%)					*p*-Value		
	Control	0	10	20	30	40	ANOVA	Linear	Quadratic
Sourness	0	1.48 ± 0.02 ^e^	0.11 ± 0.00 ^a^	0.19 ± 0.00 ^b^	0.39 ± 0.00 ^c^	0.59 ± 0.00 ^d^	<0.001	0.093	<0.001
Bitterness	0	2.34 ± 0.01 ^d^	−0.86 ± 0.00 ^c^	−0.47 ± 0.00 ^d^	−0.99 ± 0.00 ^b^	−1.01 ± 0.00 ^a^	<0.001	0.001	<0.001
Astringency	0	0.42 ± 0.02 ^b^	0.43 ± 0.00 ^b^	0.35 ± 0.005 ^a^	0.35 ± 0.00 ^a^	1.07 ± 0.005 ^c^	<0.001	0.012	<0.001
Umami	0	1.74 ± 0.02 ^e^	0.13 ± 0.003 ^a^	0.23 ± 0.005 ^b^	0.40 ± 0.005 ^c^	0.80 ± 0.005 ^d^	<0.001	0.012	<0.001
Richness	0	0.55 ± 0.02 ^e^	0.46 ± 0.005 ^d^	0.36 ± 0.005 ^c^	0.28 ± 0.005 ^b^	0.22 ± 0.005 ^a^	<0.001	0.003	0.010
Saltiness	0	0.68 ± 0.02 ^a^	1.59 ± 0.005 ^c^	1.39 ± 0.005 ^b^	2.23 ± 0.005 ^d^	2.31 ± 0.005 ^e^	<0.001	0.161	<0.001

^a–e^ Different superscript letters within the same row mean significantly different between treatments (*p* < 0.05). SEM: standard error of the mean; TVP: Textured vegetable protein.
